# Mn-induced Fermi-surface reconstruction in the SmFeAsO parent compound

**DOI:** 10.1038/s41598-021-93625-7

**Published:** 2021-07-13

**Authors:** M. Meinero, P. Bonfà, I. J. Onuorah, S. Sanna, R. De Renzi, I. Eremin, M. A. Müller, J.-C. Orain, A. Martinelli, A. Provino, P. Manfrinetti, M. Putti, T. Shiroka, G. Lamura

**Affiliations:** 1 Dipartimento di Fisica, Università di Genova, via Dodecaneso 33, 16146 Genova, Italy; 2 CNR-SPIN, Corso Perrone 24, 16152 Genova, Italy; 3 Dipartimento di Scienze Matematiche, Fisiche ed Informatiche, Università di Parma, Parco delle Scienze, 7a, 43124 Parma, Italy; 4 Dipartimento di Fisica e Astronomia “A. Righi”, Università di Bologna, Viale Berti Pichat 6/2, 40127 Bologna, Italy; 5 Theoretische Physik III, Ruhr-Universität Bochum, 44801 Bochum, Germany; 6 National University of Science and Technology MISiS, 119049 Moscow, Russian Federation; 7 Laboratory for Muon-Spin Spectroscopy, Paul Scherrer Institut, 5232 Villigen PSI, Switzerland; 8 Department of Physics and Astronomy, Rutgers, The State University of New Jersey, Piscataway, NJ 08854-8019 USA; 9 Dipartimento di Chimica e Chimica Industriale, Università di Genova, via Dodecaneso 31, 16146 Genova, Italy; 10 Laboratorium für Festkörperphysik, ETH-Hönggerberg, 8093 Zürich, Switzerland

**Keywords:** Electronic properties and materials, Magnetic properties and materials, Phase transitions and critical phenomena, Structure of solids and liquids

## Abstract

The electronic ground state of iron-based materials is unusually sensitive to electronic correlations. Among others, its delicate balance is profoundly affected by the insertion of magnetic impurities in the FeAs layers. Here, we address the effects of Fe-to-Mn substitution in the non-superconducting Sm-1111 pnictide parent compound via a comparative study of SmFe$_{1-x}$Mn$_{x}$AsO samples with $x(\text{Mn})=$ 0.05 and 0.10. Magnetization, Hall effect, and muon-spin spectroscopy data provide a coherent picture, indicating a weakening of the commensurate Fe spin-density-wave (SDW) order, as shown by the lowering of the SDW transition temperature $T_\text{SDW}$ with increasing Mn content, and the unexpected appearance of another magnetic order, occurring at $T^{*} \approx 10$ and 20 K for $x=0.05$ and 0.10, respectively. We attribute the new magnetic transition at $T^{*}$, occurring well inside the SDW phase, to a reorganization of the Fermi surface due to Fe-to-Mn substitutions. These give rise to enhanced magnetic fluctuations along the incommensurate wavevector $\varvec{Q}_2 =(\pi \pm \delta ,\pi \pm \delta )$, further increased by the RKKY interactions among Mn impurities.

## Introduction

Electronic correlations play a crucial role in the way pnictide compounds switch their originally magnetic ground state to a superconducting one and vice versa. In this context, the controlled insertion of magnetic dopants, such as Mn, is particularly relevant. The F-*doped* LaFeAsO case from the 1111 pnictide family is paradigmatic: here, tiny amounts ($\sim 0.2$%) of manganese completely suppress the superconducting state^1,2^ and are sufficient to recover the magnetic order and the tetragonal-to-orthorhombic structural transition observed in the LaFeAsO parent compound^3–5^. Strong electronic correlations enhance the magnetic coupling between the diluted Mn ions through the Ruderman–Kittel–Kasuya–Yosida (RKKY) mechanism, an indirect exchange interaction able to magnetically correlate impurity spins separated by several unit cells. This interaction reinforces the tendency towards an antiferromagnetic order in LaFe$_{1-x}$Mn$_{x}$AsO$_{1-y}$F$_{y}$^3^. Interestingly, a partial substitution of La with smaller Y ions drives the system away from quantum criticality^2^ and restores superconductivity, thus implying that a higher chemical pressure reduces the effects of Mn magnetic correlations^6,7^.

A similar picture can be drawn also for SmFe$_{1-x}$Mn$_{x}$AsO$_{1-y}$F$_{y}$: the pure superconducting phase at $x(\text{Mn}) < 0.03$ is replaced by a crossover region at intermediate Mn values $0.03< x < 0.08$, where superconductivity coexists with a static magnetic order. After completely suppressing the superconductivity (at $x = 0.08$), an even higher Mn content seems to reinstate the natural tendency towards antiferromagnetic correlation of the Mn moments through RKKY couplings^8^. Although here a higher chemical pressure with respect to the LaFe$_{1-x}$Mn$_{x}$AsO$_{1-y}$F$_{y}$ counterpart^6^ implies weaker electronic correlations, their strength is nonetheless sufficient to enhance the inter-impurity RKKY interaction, responsible for the competition between the magnetically ordered and the superconducting phase^9^.

The case of 1111 *parent* compounds with diluted magnetic impurities, although less well studied, is particularly intriguing. Thus, in LaFe$_{1-x}$Mn$_{x}$AsO, well inside the orthorhombic phase, the Mn dilution induces an incommensurate static structure associated with a charge-density-wave instability^10^. Moreover, high resolution x-ray diffraction measurements show that diluted Mn impurities can decouple the structural ($T_{s}$) and magnetic ($T_{m}$) transitions: with $T_{m}$ decreasing faster than $T_{s}$ and the magnetic order setting in only once the orthorhombic phase is well established^11^.

As we report below, our work on SmFe$_{1-x}$Mn$_{x}$AsO suggests that here an even more intricate scenario occurs. Despite a higher chemical pressure with respect to LaFe$_{1-x}$Mn$_{x}$AsO, expected to weaken the electronic correlations, in the Sm-1111 case, they are still sufficiently strong to sustain a Mn-Mn coupling via RKKY interactions. Such magnetic coupling is able to pin the electronic charges locally, resulting in a full reorganization of the Fermi surface and the onset of an incommensurate antiferromagnetic (AF) order at low temperature, well inside the existing SDW phase.

**Figure 1:**
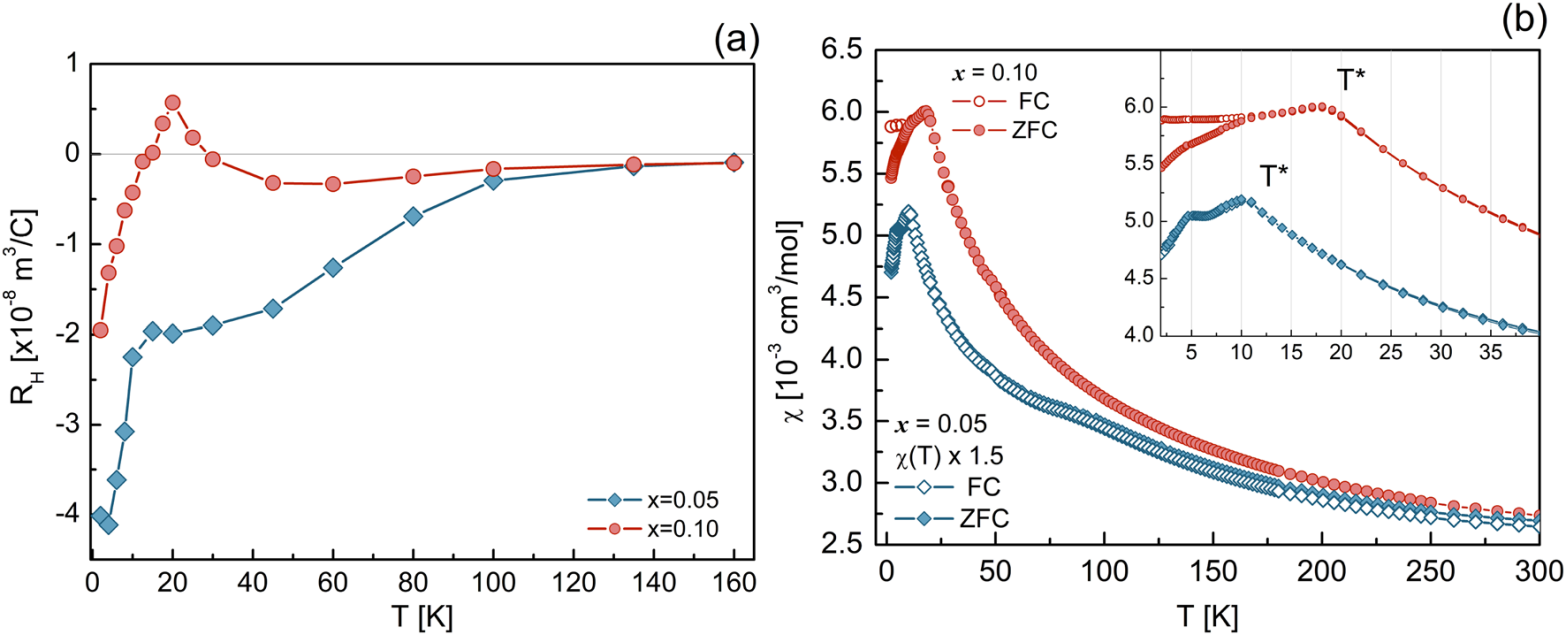
(**a**) Hall coefficient $R_\text{H}$ vs. *T* for the $x = 0.05$ (blue) and 0.10 (red) cases. See text for details. (**b**) DC magnetic susceptibility vs. temperature measured at 3 T in both zero-field-cooled (ZFC) and in field-cooled (FC) conditions. The full and open blue symbols refer to the $x=0.05$ case, while the red symbols to $x=0.10$. To facilitate a comparison, the $\chi (T)$ data for $x=0.05$ were multiplied by a factor of 1.5. The inset highlights the low-temperature features, with the cusps and changes in slope indicating the magnetic transition of the iron- and samarium ions, respectively. Here, $T^*$ indicates the magnetic anomaly induced by the Mn substitution (see text for details).

## Results

### Electrical transport and magnetic properties

As detailed in the Supplementary Information 1 (SI), we performed electrical transport measurements from 2 to 320 K, in magnetic fields up to 9 T. The temperature behavior of electrical resistivity confirms that an increased manganese content reduces $T_\text{SDW}$ and enhances the localization effects. The temperature behavior of the Hall coefficient $R_\text{H}$ in the 2–160 K range, shown in Fig. 1a for both $x=0.05$ and 0.10 cases, is particularly interesting. Above 100 K, the two $R_\text{H}$ datasets almost overlap, being both negative and negligibly small. However, upon decreasing the temperature below 100 K, they exhibit different temperature dependencies: in the $x=0.05$ case (blue diamonds), $R_\text{H}$ decreases continuously and remains negative down to low temperatures. Most importantly, $R_\text{H}$ decreases abruptly below 10 K, here defined as $T^{*}$. On the other hand, in the $x=0.10$ case (red circles), $R_\text{H}$ is almost constant down to 40 K. Then it starts increasing (changing sign at 30 K), to show a peak at $T^*=20$ K, below which it decreases sharply. Apart from the details, here related to the multiband nature of the 1111 pnictide family, the abrupt anomalies at $T^*=10$ K and 20 K strongly suggest a reorganization of the electronic bands, an effect that scales with Mn content.

The magnetic susceptibility curves, measured from 2 to 300 K at $\mu _0 H = 3$ T, both in zero-field-cooled (ZFC) and in field-cooled (FC) conditions, are shown in Fig. 1b. Generally, all curves exhibit a Curie–Weiss-like behavior and several interesting features: (1) the paramagnetic response of Sm$^{3+}$ ions dominates the overall $\chi (T)$ susceptibility, thus masking the much weaker contribution of the Fe$^{2+}$ ions, both above and below $T_\text{SDW}$^12^.(2) In the $x=0.05$ case, the ZFC and FC curves (blue filled and empty symbols) practically overlap and show a bump centered at 80 K, here corresponding to $T_\text{SDW}$. Upon cooling, an unexpected cusp appears at $T^*=10$ K, followed by a steep change in slope at 5 K, here coinciding with the Sm ordering temperature (see inset in Fig. 1b). (3) In the $x=0.10$ compound, the ZFC and FC response is similar to the previous case, except that now the magnetic SDW transition is hardly detectable. Here, too, $\chi (T)$ exhibits a cusp, but now at $T^*= 20$ K. As the temperature is further lowered, FC and ZFC depart from each other below 10 K, with a tiny change in slope still present at the Sm ordering temperature (see inset in Fig. 1b). Most importantly, the two well defined cusps in $\chi (T)$ at $T^*=10$ and 20 K [coinciding with the anomalies in the Hall coefficient $R_\text{H}(T)$] are indicative of a magnetic transition whose critical temperature scales with Mn content.

**Figure 2:**
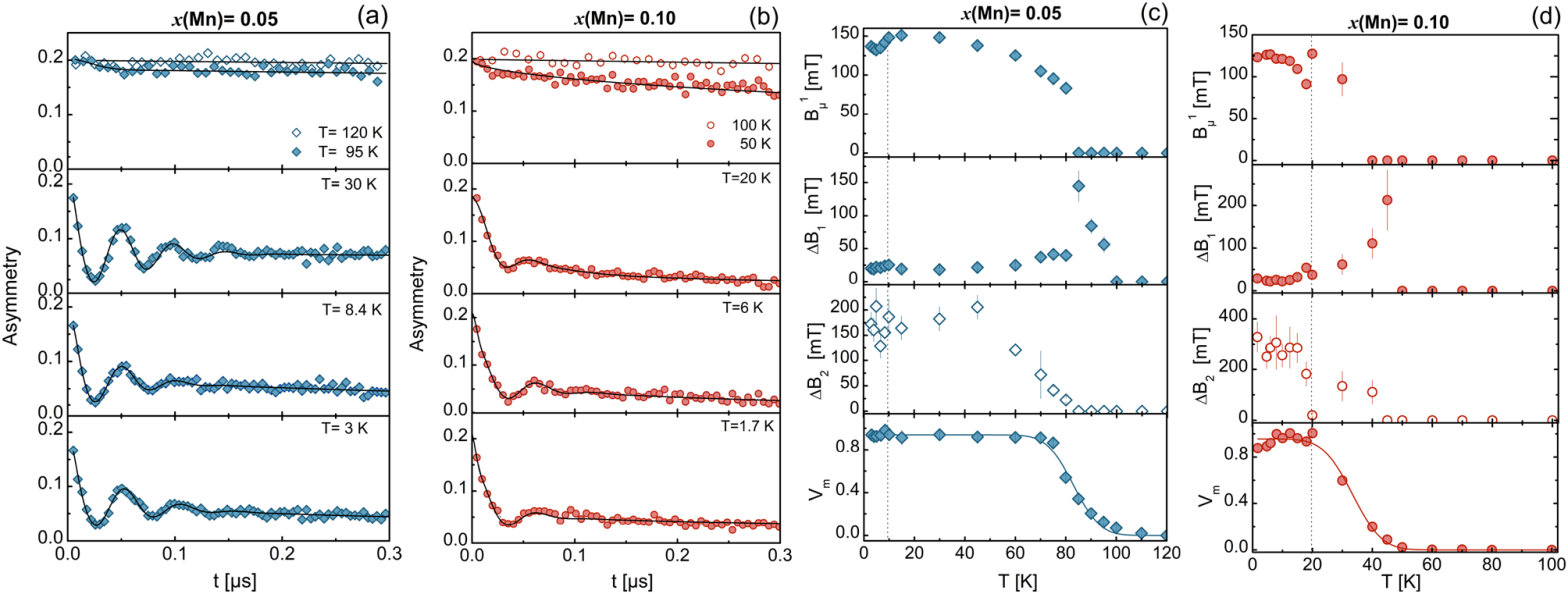
Short-time ZF-$\mu$SR asymmetries at selected temperatures for the $x = 0.05$ (**a**) and $x = 0.10$ samples (**b**). Black lines represent numerical fits by means of Eq. (SI-2) (see SI). Parameters resulting from the fits to the time-dependent asymmetry for the $x=0.05$ (**c**) and $x=0.10$ (**d**) samples. Top and medium panels report the local magnetic fields $B_\mu ^1$ and the local field widths $\varDelta B_{1,2}$ for both samples. Bottom panels show the temperature dependence of the magnetic volume fraction. Here, the lines represent numerical fits by means of an *erf* model function. The vertical dotted lines in panels (**c**,**d**) indicate $T^{*}$.

### Probing the local magnetism via zero-field $\varvec{\mu }$SR

To determine the temperature dependence of the magnetic order parameter, we carried out zero-field muon-spin spectroscopy (ZF-$\mu$SR) measurements. This technique is among the most sensitive ones for studying the local electronic properties at a microscopic scale. The time-dependent asymmetry (directly related to the spin polarization of the muon ensemble) was recorded between 1.5 and 120 K. In Fig. 2, we show the short-time asymmetry *A*(*t*) of both samples at selected temperatures. In the high-temperature paramagnetic regime ($T>T_\text{SDW}$) the two samples exhibit different behaviors: an exponential-like depolarization for $x=0.05$, characteristic of fast fluctuating electronic magnetic moments^13^, and a stretched exponential depolarization for $x=0.10$, generally valid for disordered magnetic moments (most notably for diluted spin glasses^14^) (see Methods and SI for details regarding the fit functions). In the magnetically ordered phase ($T<T_\text{SDW}$), a damped oscillation, visible only at short times (see Fig. 2a,b), indicates the onset of a static long-range magnetic order in the FeAs layers for both samples. In such cases, the best fits were obtained by using a fit model consisting of a transverse component (here the sum of a highly damped oscillating function and a fast Gaussian decay) and a longitudinal relaxing component (see SI 1). The resulting fit parameters for both Mn contents are reported in Fig. 2c,d, including the local magnetic fields $B_\mu ^{1}$ (top panels) and the Gaussian field widths $\varDelta B_{i=1,2}$ (mid-panels). Here, $i = 1, 2$ refers to the two inequivalent muon implantation sites in 1111 iron pnictides^15^.

In the $x=0.05$ case (Fig. 2c), the local field $B_\mu ^1$, probed by muons implanted at the primary sites, increases continuously as the temperature is lowered below $T_\text{SDW} = 80$ K, to reach 151 mT at 15 K. However, it decreases abruptly below $T^*=10$ K, the temperature corresponding to the cusp in $\chi (T)$ and the anomaly in $R_\text{H}(T)$. Similarly, the corresponding field width $\varDelta B_1$ (mid panel in Fig. 2c) shows two peaks: one in correspondence of $T_\text{SDW}$ and a second one at $T^*=10$ K. On the other hand, no clear precession could be detected from the muons stopped at the secondary implantation site. Here, the field width $\varDelta B_{2}$, as shown in the mid-bottom panel of Fig. 2c, starts rising at 80 K and saturates at low temperature at about 150 mT. The fit results are too noisy to reveal any relevant features at $T^*$.

**Table 1:** Magnetic ordering temperatures: $T_\text{Sm}$, $T^*$, $T_\text{SDW}$ and $\varDelta T_\text{SDW}$ width determined by dc-magnetization, Hall effect and $\mu$SR .

*x*(Mn)	$T_\text{Sm}$ (K)	$T^*$ (K)	$T_\text{SDW}$ (K)	$\varDelta T_\text{SDW}$ (K)
0.05	4.6 ± 0.3	10 ± 2	83.0 ± 0.5	8.3 ± 0.7
0.10	4.4 ± 0.6	20 ± 2	33.4 ± 0.8	7.7 ± 0.9

The last two parameters were determined from an *erf*-model fit of the *T*-dependence of the magnetic volume fraction in the $x=0.05$ and 0.10 case (see text for details).

In the $x=0.10$ case (Fig. 2d), the local $B_\mu ^1$ field is detected only below $T_\text{SDW}$, to reach $\sim 130$ mT at $T^*=20$ K. Below such temperature, we observe again an abrupt decrease of $B_\mu ^1$, followed by a progressive saturation. The corresponding field width, $\varDelta B_{1}$, is zero down to 50 K. It exhibits a pronounced peak (of 212 mT) centered at ca. 45 K, corresponding to the onset of the AF ordered state, followed by a second peak (of 54 mT) at about $T^{*}$. Finally, also in this case, no clear precessions could be detected from the muons stopped at the secondary implantation site. Here, the field width $\varDelta B_{2}$ increases with decreasing temperature and saturates at about 300 mT at 1.7 K.

ZF-$\mu$SR on polycrystalline samples gives access to the low-temperature magnetically ordered volume fraction through the measurement of the absolute value of the individual asymmetry components^16–18^. In this case, statistically 2/3 of the implanted muons precess around a local magnetic field orthogonal to their initial polarization. This precession accounts for the oscillating term in the measured asymmetry (see Eq. SI-2). The remaining 1/3 of the implanted muons probe instead a magnetic field parallel to their initial polarization. Their spins do not precess and, thus, give rise to a relaxing tail. We use this non-oscillating term to calculate the magnetic volume fraction, $V_\text{m}(T) = \frac{3}{2} \left( 1-a_{\parallel }\right)$^19^, where $a_{\parallel }=a_{L1}+a_{L2}$ and $a_{\parallel }=a_{L\text{st}}$ are the longitudinal components of the total asymmetry for the $x=0.05$ and 0.10 samples, respectively (see SM for details). In the bottom panels of Fig. 2c,d, we report the temperature dependence of the magnetic volume fraction $V_\text{m}$ for both samples. The solid lines represent numerical fits to the *erf*-model function^19^. This allows us to determine both the average magnetic transition temperature and its width (see Table 1). To summarize, we notice that: (1) at low temperatures, both samples are fully magnetically ordered. Only in the $x=0.05$ case we detect $\sim 6\%$ of a paramagnetic-like impurity. (2) As the Mn content increases, $T_\text{SDW}$ clearly decreases and broadens, with a long tail that extends up to 110 K and 60 K for $x=0.05$ and 0.10, respectively.

### Modeling the nesting of the Fermi surface

To describe the electronic structure and the magnetic instabilities in Mn-doped SmFeAsO we adopt the two-dimensional low-energy model proposed for the iron-pnictide superconductors^20–23^. It not only captures the electronic structure near the relevant high-symmetry points generated by the $d_{xz}$, $d_{yz}$, and $d_{xy}$ orbitals of iron atoms, but also allows the correct description of several magnetic instabilities in these systems. The model consists of two hole pockets near the $\varGamma$-point and two elliptical electron pockets near the *M*-point (*X* and *Y* points) of the Brillouin zone (BZ) having two-(or one) iron atoms per unit cell^24^. The detailed Hamiltonians for each of them are given in SI. By combining the descriptions near the $\varGamma$- and *M* points, one obtains the full Hamiltonian:

1 $$\begin{aligned} \mathscr {H} = \sum _\mathbf {k}\Psi (\mathbf {k})^\dagger h(\mathbf {k}) \Psi (\mathbf {k}). \end{aligned}$$

Here $\Psi (\mathbf {k})$ is the four-component spinor and $h(\mathbf {k}) = h_0(\mathbf {k}) + h_{\text {SOC}}$, where the electronic dispersion is

2 $$\begin{aligned} h_0(\mathbf {k})&= \begin{pmatrix} h_{Y,0}(\mathbf {k}) &{} 0 &{} 0 \\ 0 &{} h_{X,0}(\mathbf {k}) &{} 0 \\ 0 &{} 0 &{} h_{\varGamma ,0}(\mathbf {k}) \\ \end{pmatrix} \end{aligned}$$

and

3 $$\begin{aligned} h_{\text {SOC}}&= \begin{pmatrix} 0 &{} h_{M,\text {SOC}} &{} 0 \\ h_{M,\text {SOC}}^\dagger &{} 0 &{} 0 \\ 0 &{} 0 &{} h_{\varGamma ,\text {SOC}} \end{pmatrix} \end{aligned}$$

represents the spin-orbit coupling (SOC) interaction (see the details in SI 1).

**Figure 3:**
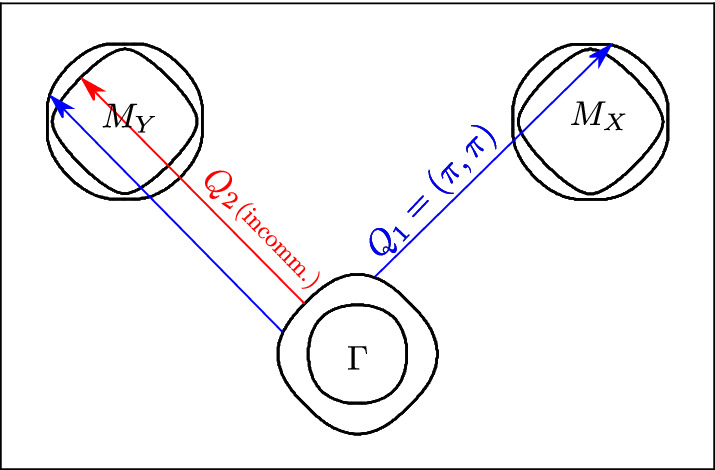
Fermi surface topology of the three-orbital model^20^, whose notation refers to the two-iron unit cell. The wave vectors $\varvec{Q}_1 = (\pm \pi ,\pi )$ and $\varvec{Q}_2 = (-\pi \pm \delta , \pi \pm \delta )$ refer to the commensurate SDW ordering wavevector of the host Fe-system and to the incommensurate order induced by the Mn impurities, respectively. *G*, $M_X$, and $M_Y$ denote the corresponding points of the BZ. Note that the outer hole pocket shows a stronger nesting with the electron pockets than the inner one.

**Figure 4:**
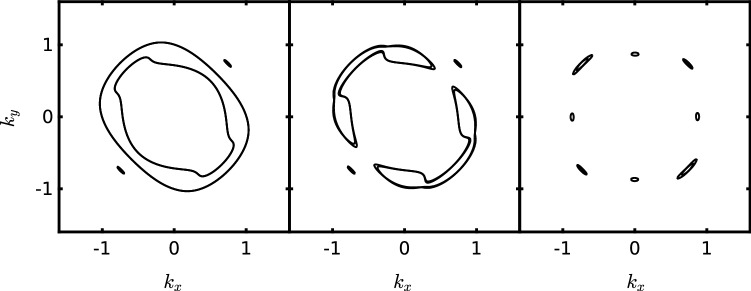
Evolution of the Fermi surface in the commensurate orthorhombic SDW state (in the folded BZ, in Å$^{-1}$ units), $\mathbf{M}_{X,c}=40$ meV (left panel), upon inclusion of the incommensurate SDW ordering induced by RKKY interactions among Mn impurities and the corresponding renormalization of the chemical potential for $\mathbf{M}_{Y,ic}/\mathbf{M}_{X,c} \approx 0.45$ (mid panel) and $\mathbf{M}_{Y,ic}/\mathbf{M}_{X,c} \approx 0.91$ (right panel). See text for details.

To describe the magnetic state of Mn-doped SmFeAsO we adopt the mean-field description, restricting ourselves to the intraorbital commensurate SDW state only (allowed by symmetry in the iron-based superconductors^21^) and neglecting the tiny interorbital contributions^25^. As there is no contribution from $d_{xy}$ orbitals near the $\varGamma$ point of BZ, only two intraorbital terms contribute to the commensurate SDW order, $\mathbf {M}_{X,c}$ and $\mathbf {M}_{Y,c}$, corresponding to the possible ordering wavevectors, $\mathbf {Q}_X = (\pi ,0)$ and $\mathbf {Q}_Y = (0,\pi )$ in the BZ with a single Fe atom per unit cell:

4 $$\begin{aligned} \mathscr {H}_{\text {SDW}}&= \mathbf {M}_{X,c}\cdot \sum _\mathbf {k}c_{yz,\mathbf {k}+ \mathbf {Q}_X,\alpha }^\dagger \varvec{\sigma }_{\alpha \beta } c_{yz,\mathbf {k},\beta } + \text {h.c.} \nonumber \\&\quad + \mathbf {M}_{Y,c} \cdot \sum _\mathbf {k}c_{xz,\mathbf {k}+ \mathbf {Q}_Y,\alpha }^\dagger \varvec{\sigma }_{\alpha \beta } c_{xz,\mathbf {k},\beta } + \text {h.c.} \end{aligned}$$

Note that, in the two-atom BZ of iron pnictides, $\mathbf {Q}_X = (\pi ,0)$ and $\mathbf {Q}_Y = (0,\pi )$ fold into the wavevector $\mathbf {Q}_1 = (\pm \pi ,\pi )$, as shown in Fig. 3. Below $T_\text{SDW}$, the onset of a commensurate orthorhombic SDW order in SmFeAsO (either $\mathbf {M}_{X,c}$ or $\mathbf {M}_{Y,c}$) breaks the rotational $C_4$ symmetry. Thus, without loss of generality, we set $\mathbf {M}_{Y,c} = 0$ and consider only a finite $\mathbf {M}_{X,c}$, with the magnetization direction along the *x*-direction, as established experimentally^3,4^. It is worth noting that here the spin-orbit coupling ($h_{\text {SOC}}$) plays an important role, since it fixes the value of the magnetic moment to be parallel to the ordering wavevector. In our case, there is only one $M_{x,c}$ component of the magnetization **M**$_{x,c}$ for the **Q**$_X$ ordering wavevector, see Refs.^21,25^. In agreement with our experimental data we assume that the same is true for the Mn-doped case, i.e., the incommensurate magnetization **M**$_{Y,ic}$ also points along $M_{Y,ic}$. Here, the role of spin-orbit coupling is to select the orientation of **M**$_{X,c}$ to be along the *x*-direction, which then automatically sets the orientation of **M**$_{Y,ic}$ to be perpendicular to **M**$_{X,c}$.

The Fermi surface topology and the resulting density of states in the AF phase are shown in Fig. 4 (left panel) and in Fig. SI-8 of SI, respectively. Mn ions, which carry a large magnetic moment $\mathbf{S}$, interact with the spin density of the quasiparticles^26^:

5 $$\begin{aligned} \mathcal {H}_\text{imp}=J_\text{ex}\sum _{\{\mathbf {i^*}\}\mu \sigma \sigma '}\mathbf{S}_{\mathbf {i^*}} \cdot \left( c_{\mathbf {i^*}\mu \sigma }^{\dagger } {\varvec{\sigma }}_{\sigma \sigma '} c_{\mathbf {i^*}\mu \sigma '}\right) , \end{aligned}$$

where $\{\mathbf {i^*}\}$ denotes the subset of atomic sites containing impurity spins. In the commensurate orthorhombic SDW state one employs the standard second-order perturbation theory with respect to $\mathcal {H}_\text{imp}$. Its application is straightforward and, after some algebra, one finds the RKKY interaction which describes the interaction between two local impurity spins at the positions *i* and *j* in the form of an *XXZ*-type effective exchange Hamiltonian (see Ref. 27). There it was shown that in the parent orthorhombic SDW state, with an ordering wavevector $\mathbf {Q}_X$ of the host system of itinerant electrons, the electron pocket originally located around the $(0,\pi )$ point of BZ and one of the hole pockets at the $\varGamma$ point remain nearly intact and are only weakly affected by the orthorhombic commensurate SDW (see Fig. 4, left panel). As a result, as shown in Fig. 3, the RKKY interaction within the parent orthorhombic SDW state appears to be stronger for the incommensurate SDW nesting wavevector $\mathbf {Q}_2=(\pi \pm \delta ,\pi \pm \delta )$, close to $\mathbf {Q}_Y$. Similarly, the interaction of quasiparticles via magnetic impurities is also enhanced along the same incommensurate SDW wavevector. This interaction can be modeled as an additional mean-field order (here h.c. denotes the Hermitian conjugate):

6 $$\begin{aligned} \mathscr {H}_{\text {SDW},ic}&= \mathbf {M}_{X,ic}\cdot \sum _\mathbf {k}c_{yz,\mathbf {k}+ \mathbf {Q}_X-\mathbf {q},\alpha }^\dagger \varvec{\sigma }_{\alpha \beta } c_{yz,\mathbf {k},\beta } + \text {h.c.}\nonumber \\&\quad + \mathbf {M}_{Y,ic} \cdot \sum _\mathbf {k}c_{xz,\mathbf {k}+ \mathbf {Q}_Y -\mathbf {q},\alpha }^\dagger \varvec{\sigma }_{\alpha \beta } c_{xz,\mathbf {k},\beta } + \text {h.c.} \end{aligned}$$

Mn impurities break the original spin-rotational and translational symmetries of the host lattice. Thus, $M_y$ may become non zero and, furthermore, **Q**$_Y$ (**Q**$_2$ in the notation of the BZ with two Fe ions per unit cell) may acquire an incommensurate value. The strength of the incommensurability is determined from the nesting properties of the residual Fermi surface in the orthorhombic commensurate (parent) AF state with non-zero $M_x$. In particular, for the parameters of the Hamiltonian we obtain an incommensurate wavevector $\mathbf {Q}_2 = (-\pi \pm \delta ,\pi \pm \delta )$ with $\delta = 0.075 \pi$, which yields a nesting of the Fermi surface inside the commensurate SDW state ($T<T_\text{SDW}$). The resulting mean-field Hamiltonian, including both the commensurate and the incommensurate SDW order, is basically a combination of Eqs. (4) and (6) (see SI 1 for the details).

**Figure 5:**
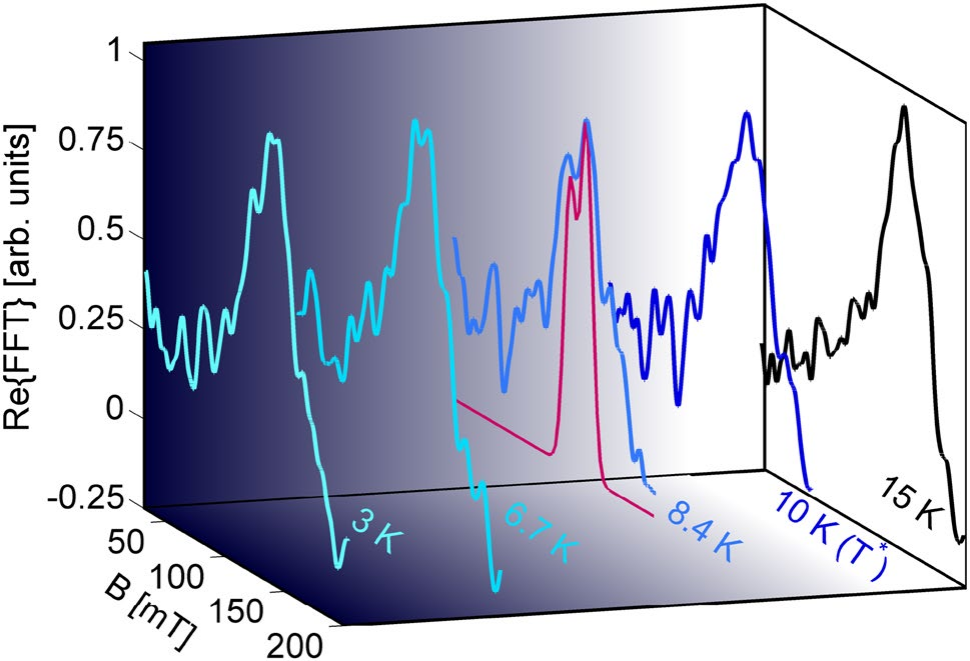
Normalized real-FT amplitude vs. $B_{\mu }$ at selected temperatures below $T_\text{SDW}$ for the $x=0.05$ case. The splitting of the main line below 10 K can be simulated numerically (red line) by considering a weighted superposition of islands with normally ordered (20%) and with tilted (80%) iron magnetic moments (0.56 $\mu _\text{B}$). Note that, despite persisting down to $T = 0$ K, the line splitting is best observed at 8.4 K, since at lower temperatures (close to 5 K) the intervening AF order of the Sm lattice interferes with Fe magnetism^28,29^.

With respect to the unperturbed case, the ordered moments are now *tilted by an angle* $\theta$, here defined as

7 $$\begin{aligned} \cos {\theta }={\mathbf{M}}_{Y,ic}/ \sqrt{\mathbf{M}^2_{X,c}+\mathbf{M}^2_{Y,ic}}, \end{aligned}$$

which represents the strength of the incommensurate SDW order. This implies that the total magnetization in the Mn-doped case, $M=\sqrt{M_{X,c}^2+M^2_{Y,ic}}$, cannot exceed the value of its parent state, here assumed equal to 40 meV, well in the range of values accepted and experimentally measured for the SDW gap^30,31^. As shown in the mid- and right panels of Fig. 4, the nesting of the residual Fermi surface of the SDW state increases upon increasing $M_{Y,ic}$, with a corresponding reduction of the density of states, even in case of small impurity concentrations (see Fig. SI-8 of SM).

**Figure 6:**
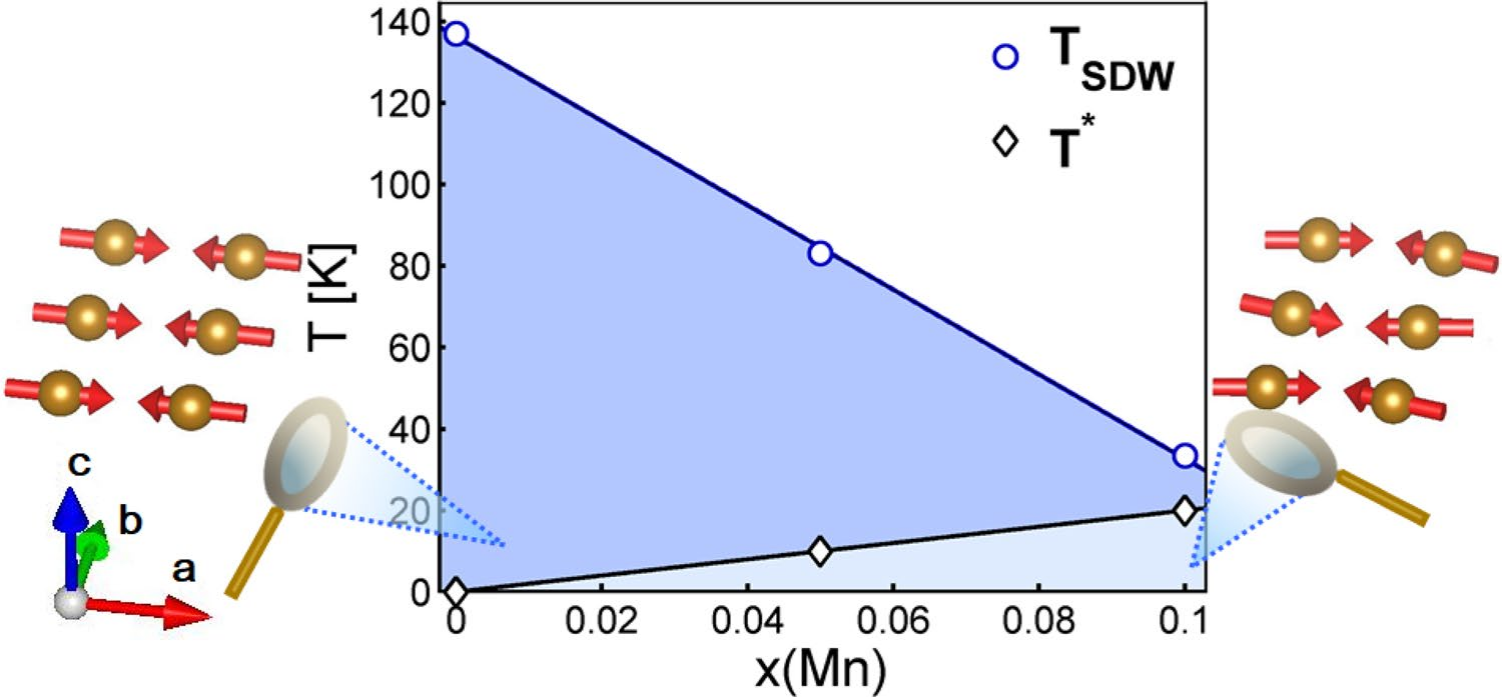
Phase diagram of SmFe$_{1-x}$Mn$_{x}$AsO, showing the commensurate ordered phase (dark-blue area) and the Mn-induced incommensurate ordered phase (light-blue area). The collinear vs. tilted arrangement of Fe moments is also sketched. Data for $x=0$ are from Ref. 13.

## Discussion

Our data clearly show that even a tiny partial substitution of Mn at the iron sites strongly affects the electronic properties of the SmFeAsO parent compound: (1) it progressively suppresses the SDW transition temperature $T_\text{SDW}$; (2) it induces a new low-temperature magnetic transition at $T^*<T_\text{SDW}$, clearly manifest in both the transport (Hall effect) and the magnetic properties (dc-magnetization and $\mu$SR). Interestingly, $T^*$ seems to scale linearly with Mn content. (3) No traces of structural transitions below the $T_\text{SDW}$ onset in the $x=0.05$ sample are evident from x-ray synchrotron radiation measurements (see SM and Ref. 5). In view of the above facts, the low-temperature electronic ground state ($T<T^*$) can be conceived to host an *additional incommensurate AF ordering* of the iron moments, mediated by the RKKY interaction between Mn ions located in the partially substituted Fe sites. Hence, the Mn ions not only interact among themselves, but they also induce changes in the Fe electronic system. This further correlation among Fe$^{2+}$ ions is responsible for the *extra nesting* of the Fermi surface in the SDW state, as shown in Fig. 4, demonstrating that even small amounts of impurities affect significantly the density of states (Fig. SI-8 in SI). Such Fermi surface reconstruction is, therefore, responsible for the low-*T* increase of the resistivity and the sizable transition at $T^*$ in the Hall effect, as reported in Figs. SI-4 in SI and 1, respectively. The additional incommensurate AF-order can be described by a small tilt angle $\theta$ of the iron moments with respect to the standard magnetic-stripe direction. Our $\mu$SR results contain the experimental signature of such effect. To illustrate this, we focus on the $x=0.05$ ZF-$\mu$SR data and consider the temperature evolution (below $T_\text{SDW}$) of the Fourier transform (FT) of the time-dependent muon-spin polarization $\mathcal {F}\{A(t)\}(\omega )$, with $\omega =\gamma _{\mu }B_{\mu }$. Here, the amplitude of the real FT signal is proportional to the local-field distribution *P*(*B*) at the two inequivalent muon sites, located in the FeAs and SmO layers, respectively. In the pristine compound (not shown), the FT is dominated by a single peak at 170 mT ($\sim 23$ MHz), corresponding to the field probed by muons implanted in the most populated site, located in the FeAs layers. Below the Sm AF-ordering at $\sim 5$ K, also two satellites appear at $\sim 110$ and $\sim 236$ mT (15 and 32 MHz), respectively^15^. This picture, valid for pure SmFeAsO, changes upon atomic substitutions (either in the FeAs or SmO layers), with the now reduced iron-moment value being reflected in a lowered and broadly distributed frequency of the main $\mu$SR peak. In addition, below the Sm ordering temperature, the two satellites become so broad as to be undetectable. Figure 5 shows the normalized muon-polarization FT spectra at selected temperatures below $T_\text{SDW}$. At 15 K a single broad peak, centered at 125 mT, is observed. As the temperature decreases, this peak narrows and progressively shifts to higher fields (up to 150 mT at $T^* = 10$ K). Below $T^*$, two key features appear: $B_{\mu }$ decreases slightly (see also Fig. 2c) and, more interestingly, a reproducible splitting appears. Despite its small value, such splitting is indicative of the tilt of Fe moments, i.e., of their new Mn-induced incommensurate ordering.

Indeed, by calculating the expected local field $B_{\mu }$ for muons implanted in the FeAs layers with a small tilt of the reduced Fe magnetic moments ($\sim 0.56$ $\mu _\text{B}$) we can reproduce the experimentally observed splitting (Fig. 5). Here, we simulate the case of a magnetic phase resulting from a patchwork of domains, with the standard AF order occupying 20% of the volume and the rest of domains containing Fe moments tilted by $3^{\circ }$ with respect to the standard alignment. The outcome of such calculation, in qualitative agreement with the data, is shown in Fig. 5 (red line). This patchwork scenario justifies also the existence of percolative paths, indicative of the further nesting of the Fermi surface below $T^{*}$ and of the small splitting observed by ZF-$\mu$SR.Therefore, we suggest that the Fe-to-Mn substitution pins regions with slightly canted Fe magnetic moments.

We summarize our findings in Fig. 6: in the SmFeAsO parent compound, small amounts of magnetic impurities not only correlate among themselves through RKKY interactions, but also cause a further nesting of the Fermi surface well inside the commensurate SDW state, thus inducing an additional incommensurate AF order of the Fe electronic system (light-blue area). In a broader perspective, the dilution of magnetic impurities in the parent compounds of iron-based superconductors can have subtle, yet very interesting effects, well illustrated here by the SmFeAsO case.

## Methods

### Sample preparation

The samples were prepared using pure elements and chemical reagents of commercial products, with weight purities of 99.9% for Sm, 99.5% for Fe, 99.999% for As, and 99.99% for Fe$_2$O$_3$ and MnO$_2$. Polycrystalline samples with a nominal composition SmFe$_{1-x}$Mn$_{x}$AsO ($x = 0$, 0.05, 0.010) were synthesized via a two-step solid-state reaction. In the first step SmAs was synthesized and then used as a precursor; fine Sm turnings and small As chips were sealed under vacuum in a Pyrex tube, heated up and treated at $560\,^{\circ }$C for three days in a resistance furnace. In the second step, the quaternary SmFe$_{1-x}$Mn$_{x}$AsO oxypnictide was synthesized. Stoichiometric amounts of SmAs, Fe, Fe$_2$O$_3$ and MnO$_2$ were well blended and ground together, so as to get a homogeneous mixture, which was then pressed into pellets of 10 mm in diameter (and a mass of $\simeq 2$–3 g) by using a hydraulic press. The pellets, sealed in outgassed Ta crucibles under Ar atmosphere, were closed under vacuum in a SiO$_2$ tube and subjected to the synthesis reaction and sintering in a resistance furnace (1200 $^{\circ }$C for 4 days); then slowly cooled down to room temperature.

### Transport and magnetometry measurements

The transport properties of all the samples were investigated from 2 to 320 K in magnetic fields up to 9 T by using a Physical Property Measurement System (PPMS, Quantum Design). To characterize the macroscopic magnetic properties of the Mn-substituted samples, we carried out both isothermal dc magnetization and dc susceptibility measurements. The isothermal magnetization (not shown) is linear in magnetic field both above and below $T_\text{SDW}$, as expected for a paramagnet and an antiferromagnet, respectively. More importantly, it proves the absence of any diluted magnetic impurities, as confirmed also by $\mu$SR results (see below).

### Zero-field $\varvec{\mu }$SR

The ZF-$\mu$SR measurements were performed in a rotated spin configuration on both the $x=0.05$ and 0.10 samples at the GPS and Dolly spectrometers of the S$\mu$S facility at the Paul Scherrer Institute, Villigen, Switzerland. The relatively large sample dimensions (ca. 8 mm diameter and 2 mm thickness) and the use of veto counters provided a good signal-to-noise ratio, hence ensuring that the collected data were only due to muons stopped in the samples. See the SI 1 for all the details regarding the employed model functions and the resulting fit parameters.

## Supplementary Information

Supplementary Information.

## Data Availability

The data that support the findings of this study are available from the corresponding author upon reasonable request.
