# Supplementary material for: Mn-induced Fermi-surface reconstruction in the SmFeAsO parent compound
Source: Sci Rep. 2021 Jul 13;11:14373. doi: 10.1038/s41598-021-93625-7 (PMC8277866; doi:10.1038/s41598-021-93625-7)
Supplement: Supplementary file 1 — Supplementary Information. [file 41598_2021_93625_MOESM1_ESM.pdf]

Supplementary Information: Mn-induced Fermi-surface reconstruction in the SmFeAsO parent compound

M. Meinero,[1,2] P. Bonfà,[3] I. J. Onuorah,[3] S. Sanna,[4] R. De Renzi,[3] I. Eremin,[5] M. A. Müller,[5] J.-C. Orain,[6] A. Martinelli,[2] A. Provino,[7,2] P. Manfrinetti,[8,2] M. Putti,[1,2] T. Shiroka,[6,9] and G. Lamura[2,*]

[1]*Dipartimento di Fisica, Università di Genova, via Dodecaneso 33, I-16146 Genova, Italy*
[2]*CNR-SPIN, Corso Perrone 24, I-16152 Genova, Italy*
[3]*Dipartimento di Scienze Matematiche, Fisiche ed Informatiche, Università di Parma, Parco delle Scienze, 7a, I-43124 Parma, Italy*
[4]*Dipartimento di Fisica e Astronomia "A. Righi", Università di Bologna, Viale Berti Pichat 6/2, I-40127 Bologna, Italy*
[5]*Theoretische Physik III, Ruhr-Universität Bochum, D-44801 Bochum, Germany*
[6]*Laboratory for Muon-Spin Spectroscopy, Paul Scherrer Institut, CH-5232 Villigen PSI, Switzerland*
[7]*Department of Physics and Astronomy, Rutgers, the State University of New Jersey, Piscataway, NJ 08854-8019, USA*
[8]*Dipartimento di Chimica e Chimica Industriale, Università di Genova, via Dodecaneso 31, I-16146 Genova, Italy*
[9]*Laboratorium für Festkörperphysik, ETH-Hönggerberg, CH-8093 Zürich, Switzerland*

(Dated: July 5, 2021)

We provide additional information on structural and transport measurements and further details on μSR data analysis and on the theoretical model.

STRUCTURAL PROPERTIES

The $SmFe_{1-x}Mn_xAsO$ samples were examined via x-ray powder diffraction using both a Guinier-Stoe camera [Cu Kα_1 radiation, with Si as internal standard, $a = 5.4308(1)$ Å] and a Philips diffractometer (Co Kα radiation). The former was used to obtain accurate lattice parameters (determined via least-squares methods) and the latter for the Rietveld structural refinement (2θ range 15–110°, with 0.02° steps). The Rietveld structural refinements were performed by using the FULLPROF program [1]. The analyzed samples resulted mostly in a tetragonal ZrCuAsSi-type phase ($tP8$, $P4/nmm$), with only a few percent of Sm_2O_3 as an extra phase (3–6 wt.%). As an example, in Fig. SI-1 we report the Rietveld refinement for the $x = 0.10$ case. The lattice parameters for the $SmFe_{1-x}Mn_xAsO$ compounds with $x = 0$, 0.05, 0.10 are summarized in Table I and plotted as a function of Mn content in Fig. SI-2. Both lattice parameters a and c increase with increasing Mn content, reflecting the larger ionic volume of Mn^{2+} with respect to Fe^{2+} [2]. Figure SI-3 shows a preliminary XRPD analysis of the $x = 0.05$ sample carried out at the high-resolution, high-intensity ID22 beamline of the European Synchrotron Radiation Facility (ESRF, Grenoble, France) above (15 K) and below (8 K) the critical temperature T^*. Remarkably, incommensurate satellite peaks are observed at both temperatures (see inset), indicating a structural modulation characterizing the low-T orthorhombic crystal structure. Similar satellite peaks were also observed in the analogous La(Fe,Mn)AsO system [3] and ascribed to the presence of a charge-density-wave instability.

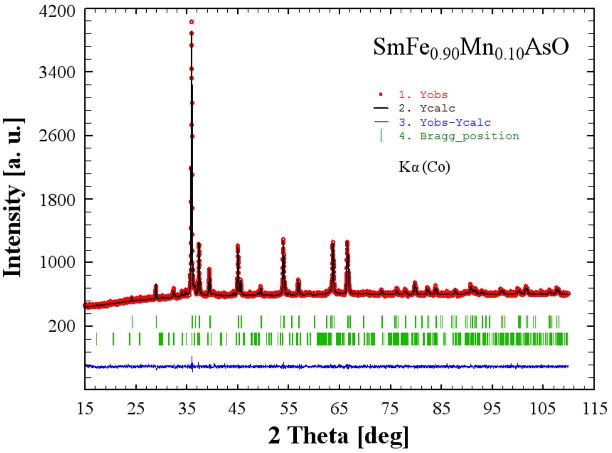

Fig. SI-1 X-ray powder pattern (red dots) and Rietveld refinement profile (black line) for the $SmFe_{0.90}Mn_{0.10}AsO$ sample (Co Kα radiation). The lower blue line is the difference between observed and calculated data; the Bragg angle positions are shown by green vertical bars for both the tested sample (upper row) and the Sm_2O_3 phase (lower row). Refinement parameters: $R_B(SmFe_{0.90}Mn_{0.10}AsO) = 1.6\%$, $R_B(Sm_2O_3) = 7.4\%$, $R_{wp} = 14.0\%$, and $\chi^2 = 1.4$.

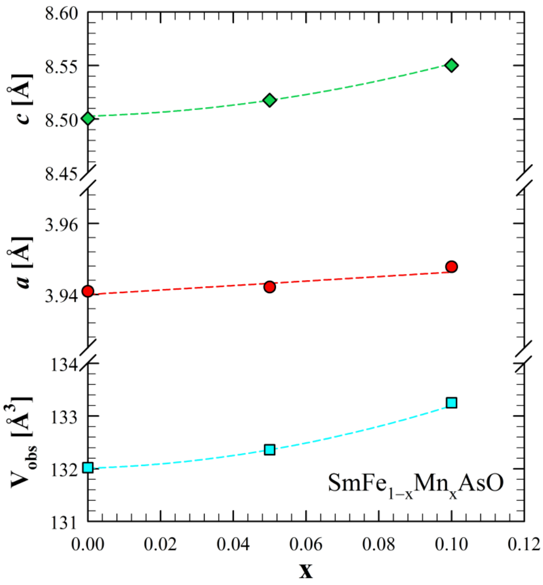

Fig. SI-2 Evolution of the $SmFe_{1-x}Mn_xAsO$ lattice parameters with Mn content ($x = 0$, 0.05, 0.10).

Table I. Lattice parameters of $SmFe_{1-x}Mn_xAsO$ vs. Mn content for $x = 0$, 0.05, and 0.10 (ZrCuAsSi-type, $tP8$, $P4/nmm$).

x (Mn)	a [Å]	c [Å]	V [Å^3]
0	3.9409(4)	8.501(2)	132.02(6)
0.05	3.9421(3)	8.518(2)	132.36(5)
0.10	3.9478(4)	8.550(1)	133.25(5)

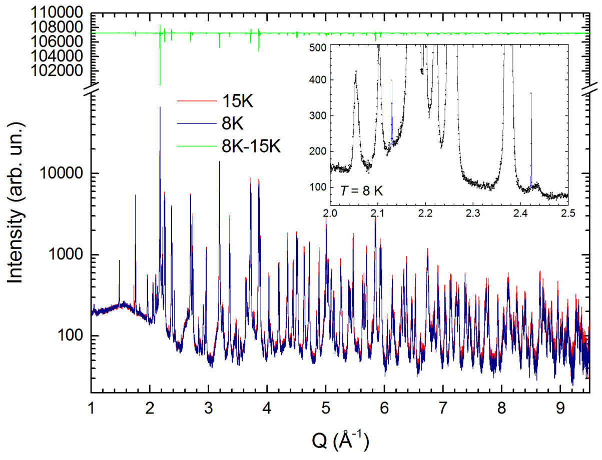

Fig. SI-3 Superposition of the synchrotron XRPD data collected above and below T^*. To enhance the low-intensity peaks, the intensities are reported in a logarithmic scale. The upper part shows the difference curve, where changes are only associated to thermal expansion and/or peak-intensity variation (i.e., no new peaks at lower T). The inset shows the magnified pattern collected at 8 K, where the incommensurate satellite peaks are highlighted in blue (the same peaks are also observed at 15 K).

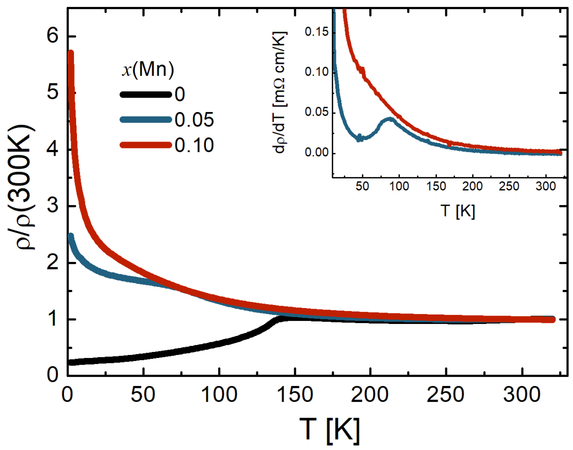

Fig. SI-4 Normalized resistivity vs. temperature in the 2–320 K range for SmFeAsO (black), $SmFe_{0.95}Mn_{0.05}AsO$ (blue) and $SmFe_{0.90}Mn_{0.10}AsO$ (red). Inset: Temperature derivatives of $\rho(T)$ for the $x = 0.05$ and $x = 0.10$ case, respectively.

TRANSPORT PROPERTIES

Electrical resistivity vs temperature Figure SI-4 shows the normalized resistivity $\rho(T)/\rho(300\,\mathrm{K})$ of the two doped samples ($x = 0.05$ and 0.10). For comparison we report also the resistivity dataset taken on the undoped compound ($x = 0$). As expected, the normalized resistivity of the $x = 0$ sample (black curve in Fig. SI-4) decreases upon cooling, exhibiting a clear anomaly at the spin density wave (SDW) transition temperature $T_{\mathrm{SDW}} \simeq 150\,\mathrm{K}$ [4, 5]. Below T_{SDW}, $\rho(T)$ decreases much faster, reaching 1.8 mΩcm at 2 K. Upon Mn doping, T_{SDW} is progressively reduced, while $\rho(T)$ increases upon cooling and shows only minor anomalies. The latter are more evident if one plots the derivative $\mathrm{d}\rho(T)/\mathrm{d}T$ vs. temperature (see inset in Fig. SI-4). Thus, in the $x = 0.05$ case, we observe a broad peak centred at about 80 K. The shape of such anomaly suggests that it represents T_{SDW}, the magnetic ordering temperature of the FeAs layers [4]. In the $x = 0.10$ case, a small change in slope at ca. 25 K suggests again the occurrence of a magnetic transition.

Table II. Room-temperature and residual resistivities of $SmFe_{1-x}Mn_xAsO$ samples. The significant increase of the latter reflects the enhanced electron localization at higher Mn content.

x(Mn)	ρ(2 K) [mΩcm]	ρ (300 K) [mΩcm]
0	1.8	7.5
0.05	15.2	6.2
0.10	42.7	7.5

The prominent increase in resistivity with decreasing temperature, most likely reflects the*weak localization effects* occurring in the Mn-substituted samples. In particular, the residual resistivity at 2 K is about 15.2 mΩcm for $x = 0.05$ and almost twice as large in the $x = 0.10$ case. As shown in Fig. SI-4, the increased scattering effects due to Mn are evident also in the normalized resistivity curves. The resistivity values of all samples, measured at $T = 2$ K and 300 K, are summarized in Table II.

Experimental details on the Hall-effect measurements
Figure SI-5 shows the transverse resistivity ρ_{xy} for the $x = 0.05$ (a) and $x = 0.10$ (b) samples as a function of the magnetic field B up to 9 T, applied perpendicular to the electric current direction for selected temperatures in the range 2–160 K. In order to remove spurious magnetoresistance components (ρ_{xx}), generally due to contact misalignments, $\rho_{xy}(B)$ was determined by extracting the antisymmetric component of the field-reversed transverse voltage V_{xy}:

$$\rho_{\mathrm{xy}}(T) = t \cdot \frac{V_{xy}(+B) - V_{xy}(-B)}{2 \cdot I}, \qquad \text{(SI-1)}$$

where t is the thickness of the samples (t ~1.5 mm) and I the applied current. The Hall coefficient R_H was subsequently derived as the slope of the linear fits to $\rho_{xy}(B)$. In the $x = 0.05$ case we performed a linear fit of $\rho_{xy}(B)$ up to 9 T, with ρ_{xy} being linear across the entire temperature- and field range. In the $x = 0.10$ case, instead, we limited the fits to 3.5 T, above which ρ_{xy} is not anymore linear in field (see inset in Fig. SI-5b).

ZERO-FIELD μSR

The analysis of the ZF-μSR time-dependent asymmetry data was performed by using the following fitting function:

$$A_{\mathrm{ZF}}(t) \;=\; \sum_{i=1}^{2}\Big[a_{T_i}\, f_i(\gamma_\mu B_\mu^i t)\, D_{T_i}(t) + a_{L_i}\, D_{L_i}(t)\Big]. \qquad \text{(SI-2)}$$

Here B_μ^i is the magnetic field at the i-th implanted muon site and $\gamma_\mu = 2\pi \times 135.53$ MHz/T is the muon gyromagnetic ratio; a_{T_i} and a_{L_i} refer to muons probing local fields in the transverse (T) or longitudinal (L) directions with respect to the initial muon-spin polarization. The coherent

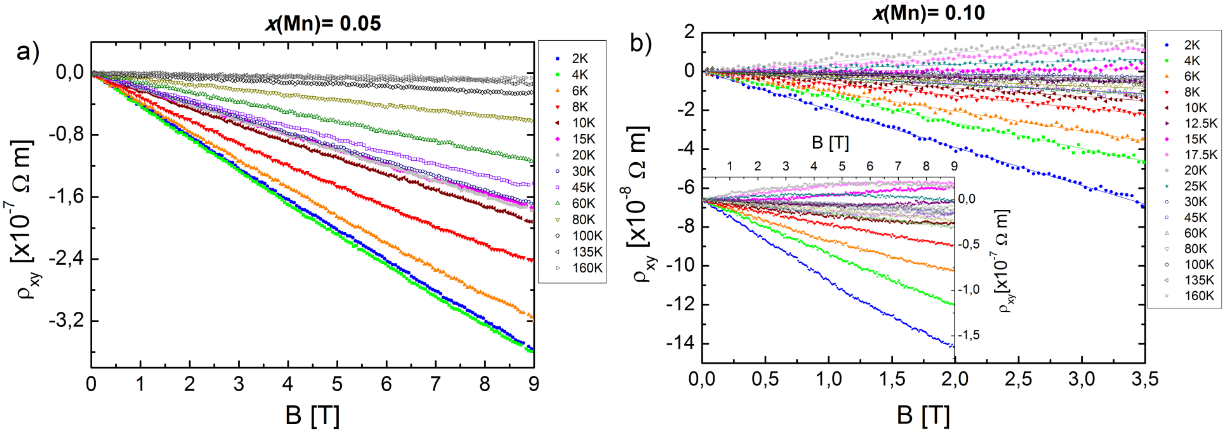

Fig. SI-5 Transverse electrical resistivity ρ_{xy} at selected temperatures in the range 2–160 K for the $x = 0.05$ sample — up to 9 T (a) and the $x = 0.10$ sample — up to 3.5 T (b). The continuos lines are linear fits to the data. Inset: ρ_{xy} for the $x = 0.10$ sample up to 9 T.

precession of muons is taken into account by the $f(\gamma_\mu B_\mu^i t)$ function, whereas $D_{T_i}(t)$ and $D_{L_i}(t)$ functions represent the precession damping: $D_{T_i}(t)$ reflects the static distribution of local magnetic fields (T) and $D_{L_i}(t)$ represents the effects of dynamical relaxation processes (L). Finally, the sum over i takes into account the two inequivalent muon implantation sites in 1111 iron pnictides. Of these, the most populated one ($i = 1$) is located near the FeAs layers, whereas the least populated one ($i = 2$) is near the SmO layers [6].

In the high-temperature paramagnetic regime the two samples exhibit different behaviors. A comparison is shown in Fig. SI-6, where the muon-spin polarization $P(t)$ (i.e., the normalised asymmetry) is plotted at selected temperatures. In the $x = 0.05$ case, for temperatures above 80 K, $P(t)$ is best described by an exponential relaxation function $D_L(t) = e^{-\lambda_L t}$, which suggests the presence of fast fluctuating electronic magnetic moments. The longitudinal relaxation rate is about 0.10 μs^{-1}, in agreement with existing data for Sm-1111 pnictides [7–9]. Below 80 K a strong depolarization due to fluctuating Sm^{3+} moments (see below) is expected [7–9]. However, the concomitant onset of the Fe^{2+}-related SDW order at the same temperature makes it difficult to disentangle the two effects. The scenario in the $x = 0.10$ case is a bit more complicated. As expected, muon spins undergo a strong depolarization below 80 K (see Fig. SI-6), likely unrelated to the Fe^{2+} SDW order, here occurring well below 50 K. Incidentally, as the temperature decreases, the

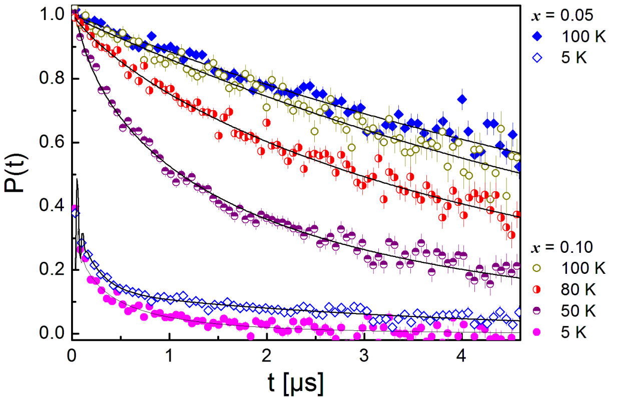

Fig. SI-6 Time-dependent ZF-μSR polarization at selected temperatures for the $x = 0.05$ and 0.10 samples, shown over the full 5-μs measurement time window.

depolarization rate becomes higher than that observed in the $x = 0.05$ case or in F-doped Sm-1111 samples [7]. In fact, by using the same fit model as in the $x = 0.05$ case, at low temperatures the slow relaxing component acquires too high values to be realistic (generally in the range up to 0.5 μs^{-1} [7–9]). This is probably the reason why the best $P(t)$ fits could be obtained with a stretched exponential model [7], generally valid for disordered magnetic moments, most notably for diluted spin glasses [10].

For $T < T_{\mathrm{SDW}}$, in the $x = 0.05$ case the best fits were obtained by assuming the transverse components to be $f_1(\gamma_\mu B_\mu^1 t) D_{T_1}(t) = \cos(\gamma_\mu B_\mu^1 t) e^{-\sigma_1{}^2 t^2/2}$ and $f_2(\gamma_\mu B_\mu^2 t) D_{T_2}(t) = e^{-\sigma_2{}^2 t^2/2}$, where $\sigma_{i=1,2} = \gamma_\mu \Delta B_{i=1,2}$ represent the field distribution widths at sites $i = 1, 2$. It is worth noting that a cosine-like oscillating term suggests the presence of a commensurate magnetically ordered phase. No significant changes of this term were detected below and above T^*, likely due to not adequate statistics at such temperatures. The relative amplitudes of these two terms resulted very similar, contrary to the usual distinction between the majority (FeAs layers) and the minority (SmO layers) muon implantation sites [9, 11, 12]. The longitudinal relaxation, instead, was modeled with two Lorentzian terms $D_{L_i}(t) = e^{-\lambda_i t}$, corresponding to a fast and a slow decay. The "slow" relaxation (with $\lambda_{L1} \simeq 0.1\,\mu s^{-1}$) accounts for the longitudinal relaxation of muons implanted in FeAs layers. The "fast" relaxation (with $\lambda_{L2} \simeq 0.1$–$4\,\mu s^{-1}$), arises from muons implanted in SmO layers and, therefore, is strongly affected by Sm^{3+} fluctuating moments. Differently from the undoped case, this last term becomes visible only below 30 K. Here, too, the $x = 0.10$ case presents some differences. Firstly, its transverse component had to be fitted by the sum of a Gaussian-damped zeroth order Bessel function ($f_1(\gamma_\mu B_\mu^1 t) D_{T_1}(t) = J_0(\gamma_\mu B_\mu^1 t) e^{-\sigma_1{}^2 t^2/2}$) and a fast Gaussian relaxation term ($f_2(\gamma_\mu B_\mu^2 t) D_{T_2}(t) = e^{-\sigma_2{}^2 t^2/2}$) whose amplitude ratio was kept fixed at all temperatures. The longitudinal fast- and slow relaxing components, instead, were merged into a single term, well modelled by a stretched exponential function $D_L(t) = e^{-(\lambda_L t)^\beta}$, as already mentioned above.

In Fig. 2a-b we show the fits of the time-dependent asymmetry at short times for both samples at selected tempera-

tures, whereas the resulting fit parameters are reported in Fig. 2c-d and discussed in the main article.

Figure SI-7a summarizes the best-fit parameters of the longitudinal-decay components of asymmetry for the $x = 0.05$ case. Below 100 K, the slow relaxation rate, λ_1 (upper panel) increases progressively, to show a broad peak at $T_{\mathrm{SDW}} = 83$ K. Upon further lowering the temperature λ_1 saturates (noisily) at about $0.2\,\mu\mathrm{s}^{-1}$. The fast relaxation rate, λ_2 (bottom panel), becomes detectable only below 30 K and rises up to $5.4\,\mu\mathrm{s}^{-1}$ at the lowest temperature. Figure SI-7b shows the longitudinal relaxation and the β parameter as a function of temperature for the $x = 0.10$ case. λ_{st} (upper panel) starts increasing as T decreases below 50 K (the onset of the magnetic transition) and peaks at $T^* = 20$ K and ~ 5 K. Unfortunately, the lack of finely spaced data does not allow us to better clarify these features. Interestingly, the stretching coefficient β is ca. 1 (Lorentzian relaxation) at high temperature. Then it decreases almost linearly with temperature down to $\beta = 0.5$, to finally show a broad peak with an onset at about 20 K.

DETAILS OF THE THEORETICAL MODEL

In the normal state, the electronic Hamiltonian near the Γ-point has the following form:

$$\mathscr{H}_\Gamma = \sum_{\mathbf{k}} \Psi_\Gamma^\dagger(\mathbf{k}) h_\Gamma(\mathbf{k}) \Psi_\Gamma(\mathbf{k}), \qquad \text{(SI-3)}$$

where $\Psi_\Gamma = \left(c_{yz,\uparrow}, -c_{xz,\uparrow}, c_{yz,\downarrow}, -c_{xz,\downarrow}\right)^T$ is the four-component spinor, while the $h_\Gamma(\mathbf{k})$ operator consists of two parts, $h_\Gamma(\mathbf{k}) = h_{\Gamma,0}(\mathbf{k}) + h_{\Gamma,\mathrm{SOC}}(\mathbf{k})$, modeling the electronic dispersion and the effects of spin-orbit coupling, respectively. The first term is given by

$$h_{\Gamma,0}(\mathbf{k}) = \begin{pmatrix} \epsilon_\Gamma + \frac{\mathbf{k}^2}{2m_\Gamma} + b k_x k_y & c(k_x^2 - k_y^2) \\ c(k_x^2 - k_y^2) & \epsilon_\Gamma + \frac{\mathbf{k}^2}{2m_\Gamma} - b k_x k_y \end{pmatrix} \otimes \sigma_0, \qquad \text{(SI-4)}$$

while the spin-orbit coupling can be expressed as

$$h_{\Gamma,\mathrm{SOC}}(\mathbf{k}) = \frac{\lambda}{2} \tau_y \otimes \sigma_z. \qquad \text{(SI-5)}$$

Here σ_i and τ_i are the Pauli matrices in the spin- and the orbital space, respectively. For the electron pockets near the X and Y point of the one-iron BZ, which are hybridized by the spin-orbit coupling in the folded two-iron BZ, the Hamiltonian has the form

$$\mathscr{H}_M = \sum_{\mathbf{k}} \left(\Psi_Y^\dagger(\mathbf{k}), \Psi_X^\dagger(\mathbf{k})\right) \begin{pmatrix} h_{Y,0}(\mathbf{k}) & h_{M,\mathrm{SOC}} \\ h_{M,\mathrm{SOC}}^\dagger & h_X(\mathbf{k}) \end{pmatrix} \begin{pmatrix} \Psi_Y(\mathbf{k}) \\ \Psi_X(\mathbf{k}) \end{pmatrix}. \qquad \text{(SI-6)}$$

Here, the spinors read $\Psi_Y = \left(c_{xz,\uparrow}, c_{xy^Y,\uparrow}, c_{xz,\downarrow}, c_{xy^Y,\downarrow}\right)^T$ and $\Psi_X = \left(c_{yz,\uparrow}, c_{xy^X,\uparrow}, c_{yz,\downarrow}, c_{xy^X,\downarrow}\right)^T$, while the dispersion is

$$h_{Y/X,0}(\mathbf{k}) = \begin{pmatrix} \epsilon_1 + \frac{\mathbf{k}^2}{2m_1} \pm a_1 k_x k_y & -i v_{Y/X}(\mathbf{k}) \\ i v_{Y/X}(\mathbf{k}) & \epsilon_3 + \frac{\mathbf{k}^2}{2m_3} \pm a_3 k_x k_y \end{pmatrix} \otimes \sigma_0, \qquad \text{(SI-7)}$$

where $v_{Y/X} = v(\pm k_x + k_y) + p_1(\pm k_x^3 + k_y^3) + p_2 k_x k_y (k_x \pm k_y)$. The spin-orbit coupling term is as follows

$$h_{M,\mathrm{SOC}} = \frac{i\lambda}{2}\left(\tau_+ \otimes \sigma_x + \tau_- \otimes \sigma_y\right). \qquad \text{(SI-8)}$$

As for the resulting mean-field Hamiltonian, which includes both the commensurate and the incommensurate SDW orders, it reads:

$$\mathscr{H} = \sum_{\mathbf{k}} \left(\Psi^\dagger(\mathbf{k}), (\Psi^\dagger(\mathbf{k}-\mathbf{q})\right) \begin{pmatrix} h(\mathbf{k}) + h_{\mathrm{SDW,com}} & h_{\mathrm{SDW,incom}} \\ h_{\mathrm{SDW,incom}}^\dagger & h(\mathbf{k}-\mathbf{q}) + h_{\mathrm{SDW,com}} \end{pmatrix} \begin{pmatrix} \Psi(\mathbf{k}) \\ \Psi(\mathbf{k}-\mathbf{q}) \end{pmatrix} \qquad \text{(SI-9)}$$

where $\Psi(\mathbf{k}) = (\Psi_Y(\mathbf{k}), \Psi_X(\mathbf{k}), \Psi_\Gamma(\mathbf{k}))$ is the total spinor, $h(\mathbf{k})$ is the combined normal state Hamiltonian and

$$h_{\mathrm{SDW,com}} = \begin{pmatrix} 0 & 0 & 0 \\ 0 & 0 & M_X \begin{pmatrix} 0 & 0 \\ -1 & 0 \end{pmatrix} \otimes \sigma_y \\ 0 & M_X \begin{pmatrix} 0 & 0 \\ -1 & 0 \end{pmatrix} \otimes \sigma_y & 0 \end{pmatrix} \qquad \text{(SI-10)}$$

,

$$h_{\mathrm{SDW,incom}} = \begin{pmatrix} 0 & 0 & M_Y \begin{pmatrix} 1 & 0 \\ 0 & 0 \end{pmatrix} \otimes \sigma_x \\ 0 & 0 & 0 \\ M_Y \begin{pmatrix} 1 & 0 \\ 0 & 0 \end{pmatrix} \otimes \sigma_x & 0 & 0 \end{pmatrix} \qquad \text{(SI-11)}$$

It is worth noting that the dimension of $\Psi(\mathbf{k})$ is 12×12, reflecting the three 4×4 matrices for each symmetry point of the Brillouin zone (Γ, X, and Y). The commensurate magnetic order, $M_{X,c}$ at $\mathbf{Q}_X = (\pi, 0)$ is contained in $\Psi(\mathbf{k})$ as off-diagonal matrix elements connecting the Γ- and the X points of the Brillouin zone, respectively. With the inclusion of the incommensurate momentum, $\Psi(\mathbf{k})$ becomes 24×24. The $M_{x,c}$ and $M_{y,c}$ values are determined by the Hubbard-Hund interactions ($M_x \propto U + J$) in the parent system and can be computed unambiguously by solving the mean-field equations. However, in the Mn-doped case, the situation is more tricky due to an additional Mn-Mn RKKY type interaction and the interaction of Mn impurities with the multiorbital host system. Therefore, the calculations become more involved. In the present manuscript we treat $M_{y,ic}$ as a parameter which minimizes the total free energy of the system by gapping the residual Fermi surface of the orthorhombic AF state. The detailed microscopic calculations are planned to be published elsewhere.

REFERENCES

* Corresponding author: gianrico.lamura@spin.cnr.it

[1] J. Rodríguez-Carvajal, Recent advances in magnetic structure determination by neutron powder diffraction, Physica B **192**, 55 (1993).

[2] P. Villars and J. L. C. Daams, Atomic-environment classification of the chemical elements, J. Alloys Compd. **197**, 177 (1993).

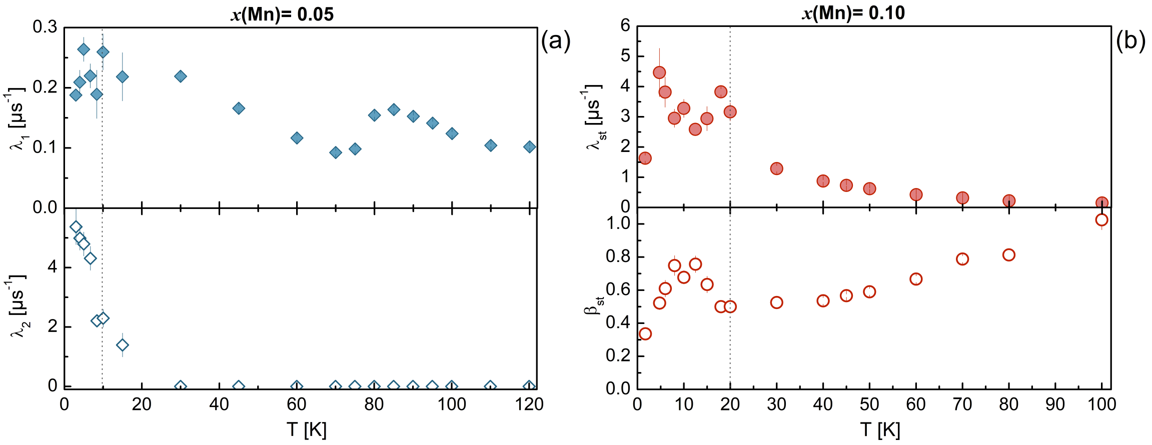

Fig. SI-7 Longitudinal relaxation rates for the $x = 0.05$ (a) and $x = 0.10$ case (b). In the first case, λ-s refer to an exponential decay model. In the second case, λ_{st} refers to a stretched-exponential model (top panel) with a stretching parameter β (bottom panel).

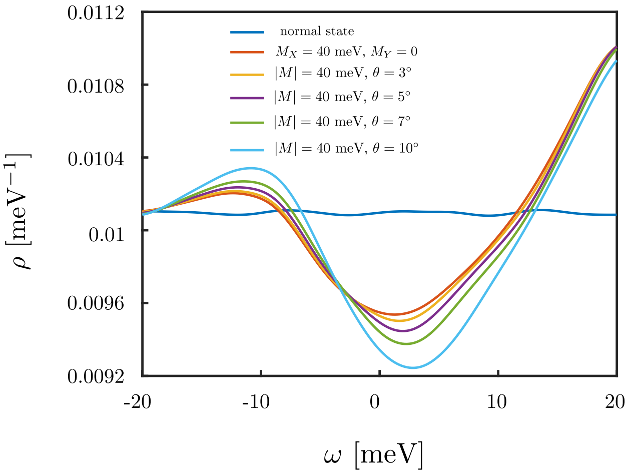

Fig. SI-8 Evolution of the density of states in the orthorhombic SDW state with $\mathbf{M}_{X,c} = 40$ meV upon inclusion of the incommensurate SDW ordering at $\mathbf{Q}_2$ induced by the RKKY interaction among Mn impurities. The angle θ defined in Eq. 7 determines the strength of the incommensurate SDW order. The total magnetization is kept constant to reveal the effect of the weak incommensurate order.

[3] A. Martinelli, P. Manfrinetti, A. Provino, A. Genovese, F. Caglieris, G. Lamura, C. Ritter, and M. Putti, Experimental evidence for static charge density waves in iron oxypnictides, Phys. Rev. Lett. **118**, 055701 (2017).

[4] A. Martinelli, M. Ferretti, P. Manfrinetti, A. Palenzona, M. Tropeano, M. R. Cimberle, C. Ferdeghini, R. Valle, C. Bernini, M. Putti, and A. S. Siri, Synthesis, crystal structure, microstructure, transport and magnetic properties of SmFeAsO and $SmFeAsO_{0.93}F_{0.07}$, Supercond. Sci. Technol. **21**, 095017 (2008).

[5] M. R. Cimberle, F. Canepa, M. Ferretti, A. Martinelli, A. Palenzona, A. S. Siri, C. Tarantini, M. Tropeano, and C. Ferdeghini, Magnetic characterization of undoped and 15% F-doped LaFeAsO and SmFeAsO compounds, J. Magn. Magn. Mater. **321**, 3024 (2009).

[6] H. Maeter, H. Luetkens, Y. G. Pashkevich, A. Kwadrin, R. Khasanov, A. Amato, A. A. Gusev, K. V. Lamonova, D. A. Chervinskii, R. Klingeler, C. Hess, G. Behr, B. Büchner, and H.-H. Klauss, Interplay of rare earth and iron magnetism in *R*FeAsO (*R* = La, Ce, Pr, and Sm): Muon-spin relaxation study and symmetry analysis, Phys. Rev. B **80**, 094524 (2009).

[7] A. J. Drew, F. L. Pratt, T. Lancaster, S. J. Blundell, P. J. Baker, R. H. Liu, G. Wu, X. H. Chen, I. Watanabe, V. K. Malik, A. Dubroka, K. W. Kim, M. Rossle, and C. Bernhard, Coexistence of magnetic fluctuations and superconductivity in the pnictide high temperature superconductor $SmFeAsO_{1-x}F_x$ measured by muon spin rotation, Phys. Rev. Lett. **101**, 097010 (2008).

[8] R. Khasanov, H. Luetkens, A. Amato, H.-H. Klauss, Z.-A. Ren, J. Yang, W. Lu, and Z.-X. Zhao, Muon spin rotation studies of $SmFeAsO_{0.85}$ and $NdFeAsO_{0.85}$ superconductors, Phys. Rev. B **78**, 092506 (2008).

[9] G. Lamura, T. Shiroka, S. Bordignon, S. Sanna, M. Moroni, R. De Renzi, P. Carretta, P. K. Biswas, F. Caglieris, M. Putti, S. Wurmehl, S. J. Singh, J. Shimoyama, M. N. Gastiasoro, and B. M. Andersen, Role of magnetic dopants in the phase diagram of Sm 1111 pnictides: The case of Mn, Phys. Rev. B **94**, 214517 (2016).

[10] R. De Renzi and S. Fanesi, Longitudinal muon relaxation in spin glasses: from the concentrated to the diluted case, Physica B **289–290**, 209 (2000).

[11] S. Sanna, R. De Renzi, G. Lamura, C. Ferdeghini, A. Palenzona, M. Putti, M. Tropeano, and T. Shiroka, Magnetic-superconducting phase boundary of $SmFeAsO_{1-x}F_x$ studied via muon spin rotation: Unified behavior in a pnictide family, Phys. Rev. B **80**, 052503 (2009).

[12] T. Shiroka, G. Lamura, S. Sanna, G. Prando, R. De Renzi, M. Tropeano, M. R. Cimberle, A. Martinelli, C. Bernini, A. Palenzona, R. Fittipaldi, A. Vecchione, P. Carretta, A. S. Siri, C. Ferdeghini, and M. Putti, Long- to short-range magnetic order in fluorine-doped CeFeAsO, Phys. Rev. B **84**, 195123 (2011).
